# Detection of Clinical Mesenchymal Cancer Cells from Bladder Wash Urine for Real-Time Detection and Prognosis

**DOI:** 10.3390/cancers11091274

**Published:** 2019-08-30

**Authors:** Bee Luan Khoo, Charlotte Bouquerel, Pradeep Durai, Sarannya Anil, Benjamin Goh, Bingcheng Wu, Lata Raman, Ratha Mahendran, Thomas Thamboo, Edmund Chiong, Chwee Teck Lim

**Affiliations:** 1 Department of Biomedical Engineering, City University of Hong Kong, 83 Tat Che e Avenue, Kowloon Tong, Hong Kong 999077, China; 2 Institut Pierre Gilles de Gennes, 75005 Paris, France; 3 Mechanobiology Institute, National University of Singapore, Singapore 117411, Singapore; 4 Department of Urology, National University Hospital, Singapore 119074, Singapore; 5 Department of Biomedical Engineering, National University of Singapore, Singapore 117583, Singapore; 6 Department of Pathology, National University Hospital, Singapore 119074, Singapore; 7 Department of Surgery, National University of Singapore, Singapore 119074, Singapore; 8 BioSystems and Micromechanics (BioSyM) IRG, Singapore-MIT Alliance for Research and Technology (SMART) Centre, Singapore 138602, Singapore; 9 Institute for Health Innovation & Technology (iHealthtech), National University of Singapore, Singapore 117599, Singapore

**Keywords:** cancer diagnosis, personalized prognosis, microfluidics, non-invasiveness, epithelial to mesenchymal transition

## Abstract

Bladder cancer (BC) is a disease that requires lifelong surveillance due to its high recurrence rate. An efficient method for the non-invasive rapid monitoring of patient prognosis and downstream phenotype characterization is warranted. Here, we develop an integrated procedure to detect aggressive mesenchymal exfoliated bladder cancer cells (EBCCs) from patients in a label-free manner. Using a combination of filtration and inertial focusing principles, the procedure allowed the focusing of EBCCs in a single stream-line for high-throughput separation from other urine components such as large squamous cells and blood cells using a microfluidic sorting device. Characterization of enriched cells can be completed within hours, suggesting a potential utility for real-time detection. We also demonstrate high efficiency of cancer cell recovery (93.3 ± 4.8%) and specific retrieval of various epithelial to mesenchymal transition (EMT) phenotype cell fractions from respective outlets of the microfluidic device. EMT is closely associated with metastasis, drug resistance and tumor-initiating potential. This procedure is validated with clinical samples, and further demonstrate the efficacy of bladder wash procedure to reduce EBCCs counts over time. Overall, the uniqueness of a rapid and non-invasive method permitting the separation of different EMT phenotypes shows high potential for clinical utility. We expect this approach will better facilitate the routine screening procedure in BC and greatly enhance personalized treatment.

## 1. Introduction

Bladder cancer (BC) is the seventh most common cancer among men worldwide and results in a yearly incidence of approximately 430,000 cases [1]. The high recurrence rate of BC means that patients often require lifelong surveillance. Currently, there are several options for BC diagnosis and follow-up and the gold standard is transurethral resection of the bladder tumor (TURBT) [2] (Table S1). TURBT is an invasive procedure where a cystoscope is inserted through the urethra into the bladder and a sample of the bladder tumor is removed by resectoscope. BC types are widely divided into two pathologic entities: Non-muscle-invasive bladder cancer (NMIBC) and muscle-invasive bladder cancer (MIBC). Upon detection, 75–85% of cases are classified as NMIBC, which is associated with a five-year survival rate of 90% [3]. However, NMIBC has a recurrence rate as high as 50–70% and 10–25% progress to muscle-invasive MIBC [2]. Therefore, BC patients still require routine monitoring for recurrence and progression after TURBT procedures. 

Liquid biopsy using blood samples has been used to isolate circulating tumor cells (CTCs) for the detection of other cancer types, namely breast, lung and colorectal cancer [4,5]. Malignant cells of BC tumors are suggested to be exfoliated spontaneously into the urine at low concentrations [6], although there is a lack of conclusive studies on the exact frequency of these exfoliated bladder cancer cells (EBCCs). Hence, EBCCs detection from urine has not been fully evaluated for use in the clinics. 

The enumeration and cytological analysis of EBCCs may serve as a complementary means for diagnosis, along with cystoscopy for the detection and surveillance of bladder cancer. However, urine cytology has low sensitivity of disease detection (0–50%) [7], and often lead to false negatives in cases of low-grade bladder tumors [8]. Moreover, none of the currently available markers used in cytology can conclusively determine the presence of BC tumors, as the markers are not unique to this disease [9]. Common markers for the detection of BC include the Nuclear Matrix Protein 22 (NMP22) and bladder tumour antigen (BTA), both of which are proteins secreted directly into the urine. The sensitivity of the NMP22 ELISA is typically 60–70% [10]. Although assays with BTA demonstrates higher sensitivity than the urine cytology test, the reported specificity was much lower [11]. Other markers such as survivin, BLCA1 and BLCA2 demonstrate higher sensitivity and specificity but were unable to provide further characterization of the cell phenotypes. Hence, the invasive and costly procedure of cystoscopy remains necessary in clinical settings. There is an urgent need to develop a procedure that can guide the characterization of EBCCs for a novel biomarker discovery, as well as the non-invasive method to better evaluate the presence and status of the disease.

One of the key caveats of using urine samples for disease management is cellular heterogeneity. A urine sample is a heterogeneous collection of different cell constituents [12] and may include white blood cells (WBCs) and red blood cells (RBCs) due to hematuria and inflammation, as well as various casts, mucus threads and squamous epithelial cells (SECs) (diameters ranging from 30 µm to 60 µm) exfoliated from the distal urethra [13]. These contaminants can severely influence the sensitivity of the cytological analysis. EBCCs also comprise various epithelial-mesenchymal transition (EMT) subpopulations, the latter a phenomenon characterized by the loss of cell-to-cell adhesion, increasing cell motility and invasiveness of cancer [14]. EBCCs may express a range of different biomarkers depending on their EMT status, of which cytokeratin (CK) and vimentin (VIM) are two protein markers that have a pivotal role in the development and progression of BC. The expression level of VIM is also associated with grade, recurrence, and progression [10,11].

Recent efforts to isolate EBCCs from urine include methods such as filtration [15,16] and immune-capture [17,18] (Table S2), but the techniques still face technical limitations. For example, filtration-based methods that involve smaller pore sizes induce shear stress that will reduce cell viability and functionality, as well as alter the morphology of cells. Other procedures based on antibody recognition may be highly specific but lack sensitivity [19]. Approaches have been developed for the sensitive and specific detection of bladder cancer [20], but the procedures are based on genomic assays which require lysis of the target cell components. Hence, a more efficient technique is highly warranted for routine and rapid clinical usage.

Here, we designed a new strategy using an EBCCs sorting (ES) device to isolate malignant EBCCs for real-time detection within hours to facilitate disease management. The procedure integrated a microfluidic assay based on the principles of inertial focusing for the high-throughput separation of EBCC subtypes based on the cell size. We aimed to develop a label-free and non-invasive procedure with the potential for EBCCs detection from urine with high robustness and sensitivity. Isolated cells were characterized with the downstream phenotypic analysis using antibodies or probes targeting established BC associated markers such as epidermal growth factor receptor (EGFR) and survivin to confirm the presence of EBCCs. Further screening with EMT markers also revealed selective enrichment of more aggressive EBCCs subtypes in one of the outlets, which could be utilized to provide insights for the clinician and assist disease management. The functional assays carried out supported the observation of different phenotypes between sorted populations.

The uniqueness of a rapid and non-invasive method permitting the separation of different EMT phenotypes bears high clinical implication. To our knowledge, no prior study has reported on the specific enrichment of mesenchymal BC cells in a label-free manner. Using the ES device, we aimed to process and characterize enriched cells on the same day, for real-time detection application and to enhance personalized treatment.

## 2. Results

### 2.1. Working Principle and Design of the ES Microfluidic Enrichment Device

In a straight channel within a microfluidic device, cells usually accumulate near the wall due to the balance between the shear-gradient lift force, directed towards the wall of the microchannel and the opposing wall effect [21]. When the fluid turns around a curve in a curvilinear channel, centrifugal forces create two secondary flows in the form of counter-rotating Dean vortices in the top and the bottom of the cross-sectional plane of the microchannel. The Dean force (*F_d_*) can be expressed as in Equation (1):

(1) $$F_{d}=3\pi \mu Ua$$

where *µ* represents the fluid viscosity, *a* being the cell diameter, and U the average velocity of the Dean Flow. Here, $U=1.8\;\times \;10^{-4}De^{1.63}$ and De is given by $\;De=Re\surd \frac{D}{2R}$. This number indicates the strength of secondary flows in these curved channels [16]. The Reynolds number is defined as Re=ρ Umax D/µ, where ρ is the fluid density, Umax (1.5 times the average velocity) is the maximum velocity of the fluid, D is the hydraulic diameter of the cross-section (Dh = 2 hw/(h + w), where h is the height and w is the width of the channel, respectively) and R is the radius of the curvature of channel.

Both the lift force and inner wall counter-effects generate another force named the inertial lift force, which depends on the distance from the wall. Ignoring the velocity variation across a cross-section, the lift force (*F_l_*) acting on the targeted particles is given by Equation (2) [16,17]

(2) $$F_{l}=\;\rho \;G^{2}\;C\;a^{4}$$

where G is the fluid shear rate (defined as G = Umax/Dh) and C is a lift coefficient that is dependent on the particle position in the channel and Reynolds number flow. 

The interplay of the lift force and Dean Drag force causes cells to migrate laterally and focus at the position where the net force equals zero. Since the magnitudes of the lift and Dean Drag forces experienced by a cell are correlated with the cell size, cells with different sizes flowing in the curvilinear micro-channels would focus at different positions on the transverse plane [17]. Large cells equilibrate at a position close to the inner wall while small cells move away from it. Here in the ES device procedure, larger cells such as the epithelial cells (diameter between 30–60 µm) will also be focused apart from the smaller bladder-exfoliated cells (diameter between 10–15 µm, Figure S4) and from the debris.

To demonstrate the precision and reproducibility of the BC cell capture by the device, cultured UMUC3 cells were serially diluted to achieve the required counts and then processed for the evaluation of cell recovery and loss rates. The design features of the sorting device could be easily modified due to the ease of fabrication using photolithography. We first screened PBS buffer spiked with UMUC3 cells using a two-outlet device. However, EBCCs from urine samples could not be sufficiently purified with the two-outlet device (Samples 1–2, Table 1) as the SECs were irregular in shape as well as highly deformable; hence, they did not attain a stable focusing condition in the device and were randomly distributed across the two outlets. Similarly, small crystals (< 4 µm) and other irregular contaminants also did not form a focused stream within the curvilinear channel and further reduced the purity of EBCCs.

**Figure 1:**
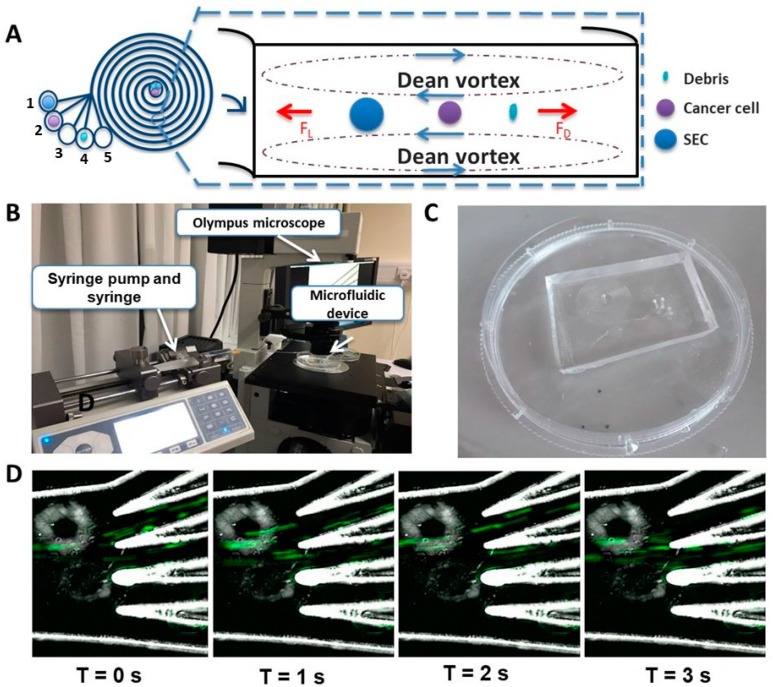
The exfoliated bladder cancer cells (EBCCs) sorting (ES) device. (**A**) The spiral microfluidic chip enriched EBCCs from urine based on the inherent hydrodynamic forces present in the microchannel that sorts cells passing through the chip based on size. Most of the EBCCs can be recovered in the second outlet. (**B**) Set-up for the high-speed capture of the flow of EBCCs in the five-outlet microfluidic device. The syringe pump introduced the sample through the device at a flow rate of 1.7 mL/min. (**C**) Actual image of the five-outlet polydimethylsiloxane (PDMS) spiral microfluidic chip. (**D**) Chronological snapshots of the flow of the UMUC3 cells through the five-outlet device. The green streaks correspond to the flow of UMUC3 cells stained with Calcein AM dye. Magnification at 20×.

We subsequently designed and fabricated a five-outlet device to allow better removal of these contaminants (Figure 1A). The channel width was 600 µm; heights were 80 µm and 130 µm at the inner and outer walls, respectively. The bifurcation was at 250 µm from the inner wall. A syringe-pump (Figure 1B) was used to introduce the sample through the polydimethylsiloxane (PDMS) microfluidic device (Figure 1C). Outlets were collected in five different tubes. In the five-outlet device, most SECs were collected in the first outlet, EBCCs entered the second outlet (Figure 1D) and other smaller containments occupied the remaining outlets. For maximum recovery, outputs from outlets 1–3 were utilized, and only outlet 2 was retrieved if a higher purity was desired. We demonstrated that the recovery rate of EBCCs in the bladder wash urine using the five-outlet device was comparable to that with the two-outlet device, and both were higher than current techniques of the EBCC capture (>75%) (two-outlet device: 85.1 ± 3.9%, five-outlet device: 80.7 ± 13.2% Figure S1).

### 2.2. Optimizing the Efficacy of EBCCs Capture

**Figure 2:**
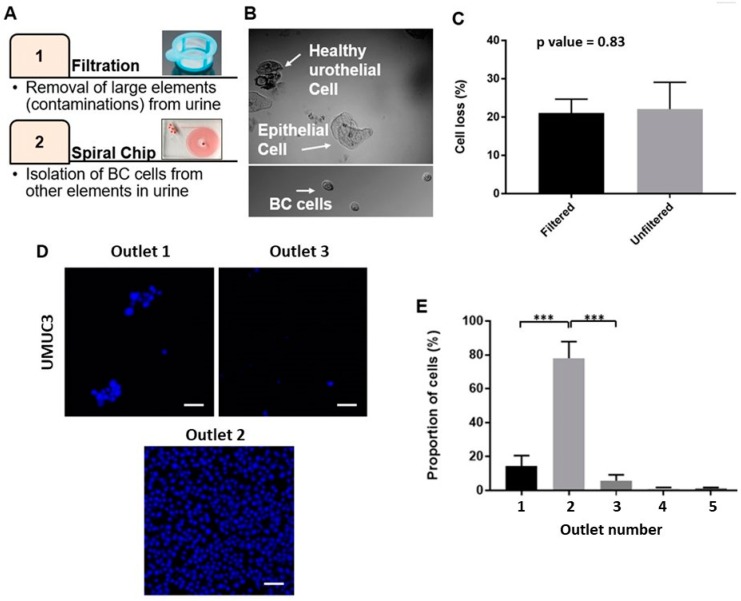
Procedure of bladder cancer cell enrichment. (**A**) The workflow of the proposed enrichment method. (**B**) Representative bright field images of larger squamous epithelial cells (SECs) (up) and smaller bladder cancer cells (down). (**C**) Difference of cell loss with filtration and without filtration (*p* value = 0.83). Data are shown as mean ± STD of triplicate wells. (**D**) Representative images of sorted UMUC3 from outlets one, two, three (from left to right) stained with Hoechst; most of the target cells go in the second outlet. The scale bar is 50 µm. (**E**) The proportion of UMUC3 cells spiked in phosphate buffer saline (PBS) found in each outlet. Data are shown as mean ± STD of triplicate wells; *** *p* < 0.001.

The urine sample is a complex pool of fluid comprising EBCCs (ranging from 11–15 µm, Figure S4A) as well as other urine components such as large squamous cells (Figure S4B), RBCs and WBCs [6]. To selectively obtain the target EBCCs, we integrated the use of filtration to remove large SECs prior to the enrichment of BC cells (Figure 2A). To reduce biofouling and shear stress, a membrane with a larger pore size was implemented to only remove SECs, irregular casts and mucus threads (Figure 2B). A residual amount of SECs might evade capture by the filter due to their high degree of deformability, but could be collected within the first outlet during device enrichment.

Cell loss during device processing is inevitable due to the adherence of cells to the channel walls, as well as due to membrane damage leading to the loss of cell integrity. Here, we reported a reduction in cell counts (22.0 ± 7.02%) between the initial spiked cell count and total cell count from all outlets. When the cell concentration was very low, such as the BC cells in the urine, cell loss became a severe problem as it prevented the detection of rare cells. To reduce this percentage of cell loss, we incorporated a surfactant coating step, using poloxamer 188 to reduce cell adherence with the channel wells. The surfactant also provided a “cell cushioning” effect by protecting the cells against shear-induced mechanical damage [22,23]. With the addition of poloxamer 188, we were able to reduce the overall cell loss from initial samples from 95% to 22%. We also evaluated if the pre-processing filtration step affects cell recovery. Imaging of the membranes after filtration confirmed the absence of the target BC cell loss in this step (Figure S2). This observation was also confirmed with the enumeration of spiked cancer cells before and after filtration. Using a sample of clinically relevant counts of spiked UMUC3 cells (e.g., 200 cells), we confirmed that the difference in terms of cell counts between the before and after filtration was not significant (*p*-value = 0.83) (Figure 2C).

We aimed to derive at a procedure that ensured the highest recovery rate and purity of EBCCs possible. To optimize the flow rate for this purpose, different flow rates between 0.5 mL/min and 2 mL/min were screened. We demonstrated that at a flow rate of 0.5 mL/min, there was no stable focusing and cells were randomly sorted into the outlets (Figure S3). When the flow rate reached 1.0 mL/min, target cells began to selectively sort themselves within the first two outlets. Between flow rates of 1.5 mL/min to 2.0 mL/min, all the target cells were well focused at the second outlet, with only a few target cells going to the first and third outlets. Hence, we derived the optimum flow rate at 1.7 mL/min, which was then used for the remaining screenings.

### 2.3. Performance of Bladder Cancer Cells Enrichment Platform

The UMUC3 average cell size was determined by imaging and analysis with Image J (Figure S4; 12.9 ± 0.83 µm). As compared to the average cell size of other blood cell constituents in urine (RBCs: 6–7 µm, WBCs: 10–13 µm), UMUC3 cells were relatively larger, which facilitated device sorting. 

To identify if cell viability was influenced by spiral processing, UMUC3 cells were spiked into 40 mL of cell-free urine samples conditioned with 0.3% poloxamer 188. The cell-free urine was obtained as the supernatant of centrifuged urine samples. The spiked sample was evenly split into two portions to compare the difference in cell viability before and after device processing. Using the Trypan Blue assay, we demonstrated that no significant difference in cell viability was observed between the pre-sorted (90.0 ± 2.5%) and sorted group (85.3 ± 2.5%) (*p*-value = 0.13; Figure S5A). We also screened for the viability of cells under exposure to various concentrations of poloxamer 188 for 3 h and confirmed that the addition of poloxamer 188 did not cause significant change to the cell viability (Figure S5B).

To mimic clinical samples, UMUC3 cells in 3 mL PBS with 0.3% poloxamer 188 were prepared for processing to evaluate the device performance. The recovery rate was calculated as:

(3) $$Cell\;recovery\;rate=\;\frac{Number\;of\;target\;cells\;in\;target\;outlet}{Total\;number\;of\;cells\;found\;in\;all\;outlets\;}\;$$

Cells collected from each outlet of the spiral microfluidic device after sorting of the spiked urine sample were stained with Hoechst in a 96-well plate and imaged (Figure 2D). The recovery rate of cells in the target outlets (innermost first three outlets) was 93.3 ± 4.8%, with most cells being captured within the second outlet (77.4 ± 6.2%) (Figure 2E), and almost no cells (2.54 ± 1.0%) found in the last two outlets. 

Urine samples may become cloudy over prolonged periods, possibly due to the accumulation of bacteria or precipitated phosphate crystals [24]. Hence, we investigated the effects of varying concentrations of the bovine serum albumin (BSA) on urine cloudiness. Urine samples were exposed to varying concentrations of BSA for 3 h prior to analysis. A microtiter analysis was utilized to check for the relative absorbance of the samples conditioned with different BSA conditions (Figure S6). The graph of the relative absorbance of each sample against the respective concentration of BSA showed that the concentration of 1.5% BSA led to a sample with the lowest relative absorbance. We subsequently added 1.5% BSA to all samples to allow the efficient processing of the images and accurate cell count of EBCCs. 

### 2.4. Detection of EBCCs from Spiked Cell Line Samples and Clinical Patients

One of the most challenging factors affecting cancer management is tumor heterogeneity. Tumor heterogeneity may occur in various forms, including shape, size, gene and protein expression. This variation in tumor phenotype remains a key caveat of cancer cell detection. For example, in the area of inertial sorting, previous studies and mathematic models theorizing these principles assume that the flowing particles are all spherical, i.e., same radius from the center in all directions [25,26]. However, inertial focusing is also influenced by other factors such as cell shape and deformability, but these effects are not yet completely understood. 

In this study, seven patients were recruited for the initial optimization of the ES device design and operations (n = 5), as well as downstream cell characterization (n = 2) (Table 1). These patients were diagnosed with different stages of BC, namely non-muscle invasive BC, muscle-invasive BC and metastatic BC. Four samples were obtained from each patient at different time points of a bladder wash procedure, where the first sample correlated to the unwashed urine samples. This is one of the approved methods of collection for urine cytology. In our study, voided urine for cytology was collected from patients during the preoperative assessment as a part of the standard of care. The preoperative voided urine cytology can act as an internal control and be used to validate and compare our device efficacy in the detection of cancer cells from patients. Hence, if the device could identify cancer cells in a patient with negative urine cytology, the results would be complementary to aid early cancer diagnosis or in the identification of early recurrences.

We aimed to investigate if the bladder wash procedure could effectively reduce the number of cancer cells present in the bladder after resection. Based on established records on the cell size of epithelial cells, we fixed the size threshold value as 30 µm to distinguish cancer cells from larger squamous epithelial cells. However, it was expected that patient cancer cells would be more heterogeneous in both their cell surface markers and biophysical properties than clinical EBCCs.

We confirmed the presence of EBCCs from samples of target outlets via histopathological staining or immunostaining (Figure S7). Histological stains were prepared and analyzed by a clinical pathologist. In terms of immunostaining, we stained sorted samples from each time point with survivin, a key marker for bladder cancer. Survivin is an apoptosis inhibitor protein expressed in the G2/M phase of the cell cycle [27] and has been reported for use as a diagnostic biomarker. Survivin is also used for the monitoring of bladder cancer as it gives high positive results particularly in low grade, early-stage disease. We also characterized isolated cells with markers associated with epithelial tumors, VIM and CK.

**Figure 3:**
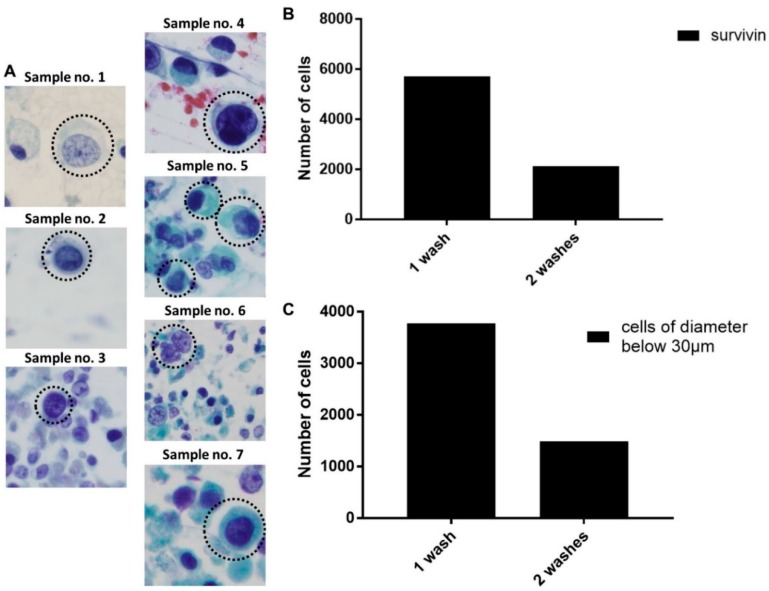
Patient bladder washes processing and bladder cancer (BC) cell counting. (**A**) Representative images of atypical EBCCs from sorted samples of each patient. (**B**) The decreasing number of survivin+ cancer cells after each washing step. (**C**) The decreasing number of cells with a diameter below 30 µm.

During our histological analysis of sorted EBCCs, we observed similar variations of atypical tumor cells in terms of physical attributes across the clinical samples (Figure 3A). This justified our use of a multi-outlet device for cell capture, to allow the collection of samples depending on the tumor status of a patient. Using the above parameters, we analyzed the clinical samples (*n* = 2) and observed a significant reduction of cancer cells after consecutive bladder wash procedures, of which 61.7 ± 1.1%. 8% of the EBCCs were removed within the first two rounds of the bladder wash procedure (Figure 3B,C). The size range of EBCCs was 60 µm^2^ and above. Samples from the two remaining time points comprised of mostly debris. Sample 7 could not be enumerated due to sample conditions. Although a feedback loop could be introduced to allow higher purity of target cells, this was not carried out in the study, as the primary EBCCs might be relatively fragile after exposure to urine conditions.

**Figure 4:**
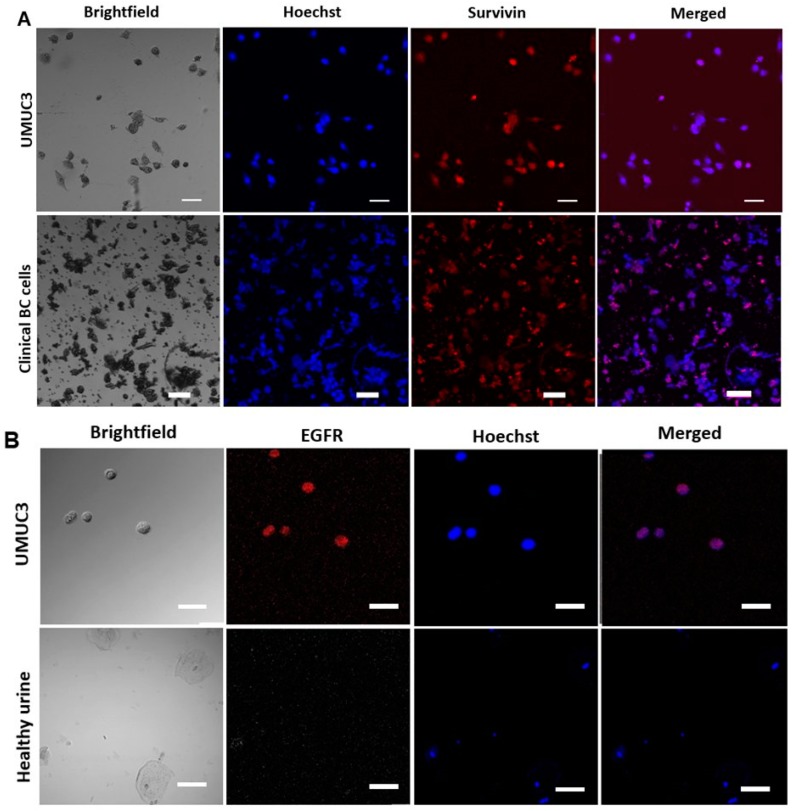
Bladder cancer markers. (**A**) Staining of control cultured UMUC3 and of clinical bladder cancer cells with survivin and Hoechst. Representative frames of bright field and merged images are shown. The scale bar is 50 µm. (**B**) Epidermal growth factor receptor (EGFR) staining on UMUC3 cells spiked in PBS. Healthy urine is provided as a control. Healthy urothelial cells are stained with Hoechst but not with EGFR. The scale bar is 50 µm.

The expression of survivin was also heterogeneous within a patient’s EBCC population (Figure 4A). We were also able to detect the variable presence of the bladder cancer-associated gene, EGFR, using the in situ fluorescence hybridization (FISH) assay (Figure 4B). The EGFR protein is related to the type 1 tyrosine kinase receptor family [28] and is important for the progression of bladder cancer. Recent studies show that EGFR is positive in more than 60% of bladder tumors [28].

### 2.5. Enhanced Recovery of Clinical Mesenchymal Bladder Cancer Cells

Cancer cells expressing variable levels of epithelial and mesenchymal markers have been widely reported [29]. During the process of EMT, cancer cells undergo various physiological changes [30]. VIM is highly expressed in mesenchymal cells and it enhances cell elastic behavior as well as protect the cell against compressive stress [31]. The EMT markers are not unique to cancer cells and are used along with bladder cancer-specific markers for analysis. 

**Figure 5:**
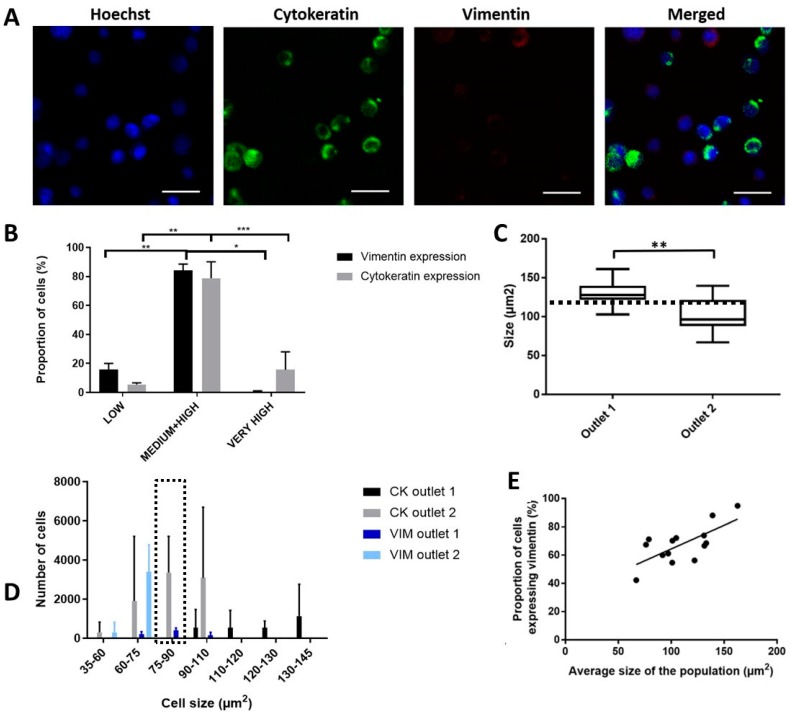
Specific enrichment of mesenchymal EBCCs. Data are shown as mean ± STD of triplicate wells. (**A**) Immunostaining of cells with Hoechst, cytokeratin (CK) and vimentin (VIM). The scale is 50 µm. (**B**) The gradient of fluorescence intensity for CK and VIM. * *p* < 0.05, ** *p* < 0.01. (**C**) Cells isolated in the first outlet have a bigger size. *p*-value = 0.003, ** *p* < 0.01. (**D**) The proportion of CK+ and VIM+ population sorted for each size. Marked region highlights cells within the range of 75–90 µm^2^. (**E**) Correlation between size and VIM expression.

Due to the changes in physiological properties between mesenchymal and epithelial cells, we believe that cells along the EMT spectrum could be sorted differently. To evaluate the presence of differential sorting between cancer cell populations, we used two cancer cell lines (UMUC3 and T24) with mesenchymal and epithelial EMT phenotypes, respectively. Cells were stained with anti-VIM and anti-CK antibodies prior to sorting with the ES device (Figure 5A). We observed a non-uniform expression of these markers even within the same cancer cell line, giving rise to a gradient of fluorescence intensity which we categorized into four levels of expression for ease of evaluation (threshold values for 1. Low expression: 1 AU, 2. Medium expression: 10 AU, 3. High expression: 30 AU and 4. Very high expression: > 30 AU; AU = Arbitrary Units) (Figure 5B). Most of the cancer cells had a medium or high expression of CK and VIM (>80%), although a small proportion had low (CK: 4.8 ± 0.57%, VIM: 14.7 ± 0.28%) or very high expression (more than 30 AU; CK: 13.3 ± 7.15%, VIM: 0.6 ± 0.9%) of CK and VIM. 

We processed a mixed sample of UMUC3 and T24 cells spiked in PBS using a 1:1 ratio. Although UMUC3 mesenchymal cells were generally smaller than the T24 epithelial cells, there was an overlapping range of similar cell size (<110 µm^2^). T24 and UMUC3 cells were pre-labelled with CK- Fluorescein isothiocyanate (FITC) and VIM-PE, respectively prior to mixing. The size of cells sorted into the first outlet was significantly larger than that for the second outlet (*p*-value = 0.002), with the cell size threshold around 100.5 ± 22.22 µm^2^ (Figure 5C). We observed that cells above the threshold that were sorted into the first outlet were all epithelial cells. However, UMUC3 and T24 cells within the overlapping cell size range (<101 µm^2^) were not sorted into the same outlets, suggesting that the sorting efficacy was influenced by the physical attributes inflicted under different EMT status (Figure 5D). Specifically, for cells within the range of 75–90 µm^2^, most mesenchymal cells (94.7 ± 4.05%) were sorted into the first outlet while all of the epithelial cells were sorted instead into the second outlet. We also confirmed that larger cells expressed a higher degree of VIM, and a significant positive correlation (R square of 0.48) between the average cell size and proportion of VIM expression could be derived (Figure 5E). Hence, the procedure can effectively select for more large mesenchymal cancer cells from specific outlets, an important subtype which is highly associated with tumor progression and worsened clinical prognosis [32].

**Figure 6:**
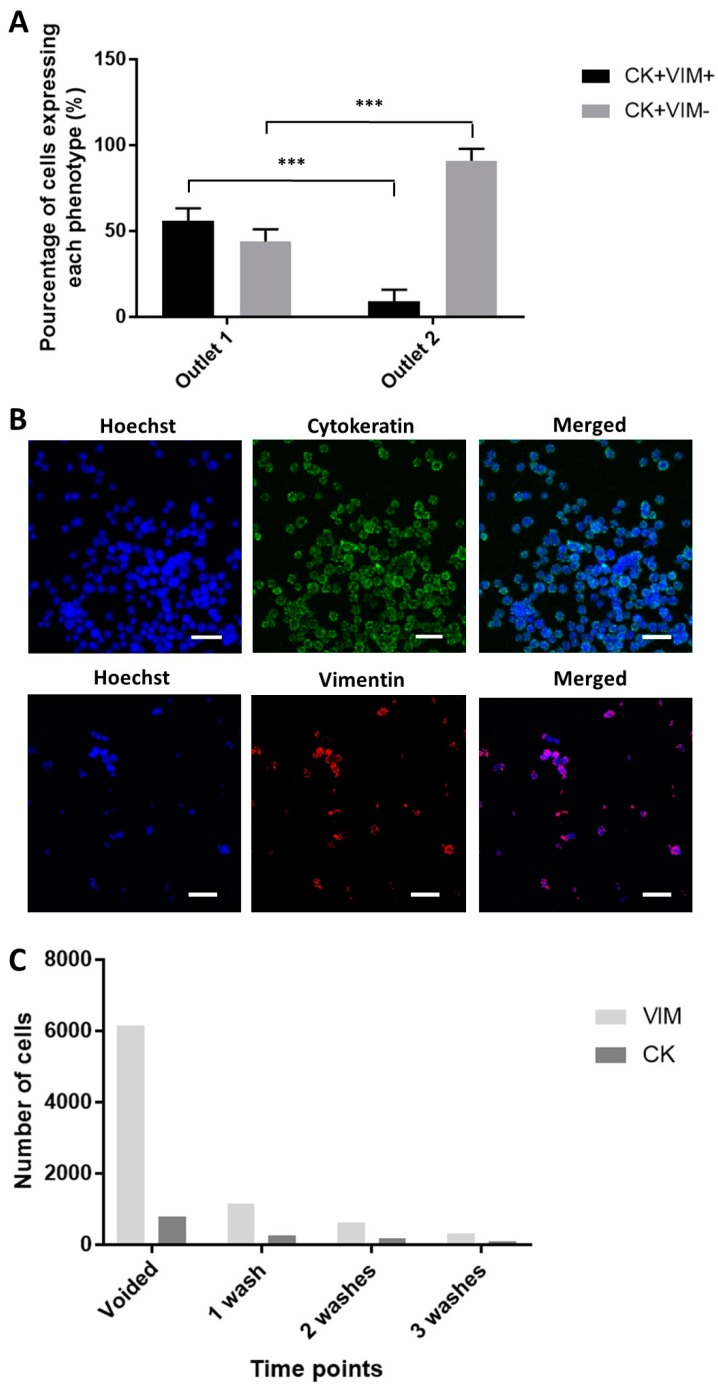
Enrichment of clinical BC cells. (**A**) Proportion of intermediate and complete epithelial to mesenchymal transition (EMT) phenotype isolated in each outlet (*p*-value = 0.0002). Data are shown as mean ± STD of triplicate wells; *** *p* < 0.001. (**B**) Positive controls of antibodies targeting for CK and VIM. The scale bar is 50 µm. (**C**) The proportional reduction of epithelial and mesenchymal cells after each bladder wash.

In clinical samples, the occurrence of a complete EMT involving the loss of all epithelial traits and concomitant gain of the full spectrum of mesenchymal attributes (as seen in certain EMT processes during embryogenesis) is rare in human carcinomas [33]. RNA in situ hybridization analysis of circulating tumor cells (CTCs) from women with breast cancer revealed that some of them express both epithelial and mesenchymal transcripts, a process named as partial EMT activation [34]. With the clinical samples (*n* = 2), we observed a similarly high degree of tumor heterogeneity in terms of EMT phenotype. Sorted samples were evaluated with EMT and bladder cancer associated markers, namely CK, VIM, and survivin. Most of the samples comprise epithelial cells, with mesenchymal cells being largely absent. However, one of the samples (Sample no. 7) had about five times more mesenchymal cells than epithelial cells. Clinical EBCCs with an epithelial-like phenotype (CK + VIM−) was mostly isolated in the second outlet (90.9% ± 7%) (Figure 6A), suggesting that large epithelial cells greater than the threshold of 101 µm^2^ was not present. UMUC3 cells, MCF7 cells and MDA-MB-231 cells were used as negative or positive controls for the antibodies (Figure 6B). The proportion of VIM+ and CK+ cell counts recovered from the device were reduced at a consistent rate after various rounds of bladder washes (Figure 6C).

To the best of our knowledge, no enrichment methods have yet to report the specific enrichment of EMT subtypes, despite the importance of identifying specific cancer cell populations in cancer progression. In addition to mesenchymal cells, the enrichment of cancer cells with the intermediate EMT phenotype is of particular interest, as they are associated with cancer stem cells, a subpopulation of cancer cells which are characterized by the capacity for self-renewal and the ability to differentiate into diverse specialized cell types [35]. In the clinical samples, we also observed cells with an intermediate EMT phenotype (CK + VIM+), which were isolated mainly in the first outlet (56.1% ± 8.99%). Overall, we demonstrate a proof of concept that the integrated sorting procedure can selectively enrich aggressive cancer cell types, which will be beneficial for drug discovery and evaluation of patient prognosis. 

### 2.6. Functional Characterization of Sorted Mesenchymal Bladder Cancer Cells

EMT is a major factor of tumor heterogeneity and results in a spectrum of tumor cell phenotypes that can aid in tumor progression in different ways. Among these subpopulations, mesenchymal cells are highly associated with low overall survival in patients [33]. These mesenchymal cells demonstrate heightened functional traits such as invasiveness and migration speeds to facilitate metastasis.

**Figure 7:**
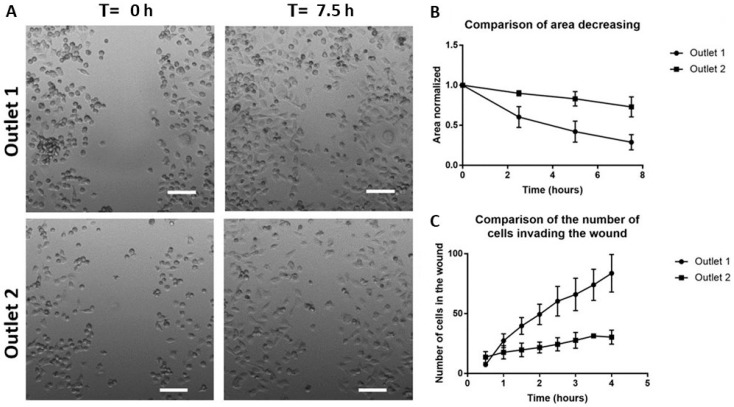
Wound closure measurements. (**A**) A scratch is made at T = 0 h and the wound healing is observed over 7.5 h. Scale bar = 100 µm. (**B**) Area decreasing over time. (**C**) An increasing number of cells invading the wound over time.

To confirm the specific enrichment of mesenchymal cells in the first outlet, we used the samples collected from the target outlets for a scratch-wound assay (Figure 7A) [36]. We first characterized the area of the wound over time and observed a faster closure of the wound area with sorted cells from outlet one (50% wound closure: 4.44 ± 1.02 h) as compared to those from outlet two (50% wound closure: >7.5 h) (14.0 ± 7.6 h) (Figure 7B,C). To avoid bias due to the difference in the size of the wound area, we also enumerated the number of cells invading the wound area and observed a higher number of cells inside the wound within the same period from samples of the first outlet (0.5 h: 7.33 ± 0.5 cells, 4 h: 83.6 ± 15.7 cells), in comparison to the second outlet (0.5 h: 13.6 ± 4.6 cells, 4 h: 30.3 ± 5.85 cells) (Figure 7C). Hence, we confirmed the specific sorting of more aggressive cancer phenotypes, an outcome which will be beneficial for clinicians in guiding diagnostic response. The differential sorting of EMT phenotypes is also highly relevant for the development of current therapies as mesenchymal phenotypes are also closely related to heightened traits of drug-resistance.

## 3. Discussion

Bladder cancer has a high recurrence rate, and errors in clinical staging as well as pathological grading of bladder cancer are frequent [37]. The main problem of an invasive therapeutic procedure for BC diagnosis and prognosis is burning of the tissue during cauterization, making it difficult to determine whether the superficial layer or stroma has been invaded, moreover invasive procedures are very painful for the patient. The development of a simple and robust platform capable of detecting BC with non-invasiveness and high sensitivity remains a challenge. Here we demonstrate the application of a non-invasive, label-free procedure using inertial focusing microfluidics for the isolation of BC cells from urine. Due to the scope of this project for demonstrating the efficacy of bladder wash procedures in the reduction of EBCC counts over time, bladder wash urine samples were utilized throughout this study. Four samples were obtained from each patient at different time points of a bladder wash procedure, where the first unwashed sample correlates to the voided urine for cytology. This is one of the approved methods of collection for urine cytology. The significance of this microfluidic sorting device is the ability to isolate intact bladder cancer cells in a label-free manner, under minimal shear stress and high sensitivity (93.3 ± 4.8%), which will allow cells to remain viable for downstream analysis, including culture. This provides unprecedented opportunities for the efficient enrichment of these rare cells, allowing easier downstream characterization and prognosis of BC. Our two-step method captures cells of interest while eliminating background cells (larger SECs and smaller leucocytes) for detection and downstream phenotype characterization.

Compared with other gold standards of diagnosis, the method we developed has the following technical merits: 1. Efficient processing of clinically relevant urine sample volumes within a short period of time (20 mL urine in 20 min); 2. high recovery, consistency, and sensitivity; and 3. viable enriched cell fraction which can be used for downstream analysis. The procedure can also be modified to remove squamous epithelial cells instead via negative selection, which will prevent the loss of bladder cancer cell clusters. Clustered cancer cells have been shown to correlate with clinical outcomes in various cancer types [38], and may be important parameters for diagnosis. Overall, the fast processing time will help to translate this enrichment platform to in-clinic and ‘point-of-care’ applications. The simplicity in the manufacturing of the spiral microchannel device and the ease in handling of the equipment and device also make this enrichment method attractive for clinical applications requiring a one-time-use operation.

The rapid enrichment procedure allows selective enrichment of mesenchymal and intermediate EMT phenotypes. EMT occurs during the progression of tumors, endowing cancer cells with increased motility and invasiveness [39]. Since mesenchymal and epithelial cells have different shapes and deformability properties [40], we were able to isolate different fractions of epithelial and mesenchymal cells into respective outlets of the device. EMT phenotypes have been widely shown to correlate with tumor-initiating capabilities [41,42]. Using EMT markers, we classified subpopulations of the sorted cells (Figure 5B) and further evaluated their metastatic properties via wound healing assays, which confirmed the heightened metastatic properties of sorted cells from the target outlet (Figure 7). The specific enrichment of these subpopulations may be valuable for the expansion of cell lines to be used in the design of new therapies targeting EMT [43,44]. The EMT is a reversible process and new strategies inducing EMT reversal are promising to suppress cancer cell migration and metastasis [45]. Moreover, accumulating evidence indicates that conventional therapies often fail to eradicate cancer cells that have entered the stem state, which is activated via EMT [46]. These cells, termed as cancer stem cells (CSCs), are a major hurdle to traditional therapies [47]. Therapies include the induction of CSCs apoptosis and induction of CSCs differentiation by interrupting signals from the microenvironment that regulate important properties like self-renewal, differentiation and apoptosis resistance. They hold great promise for cancer therapy whereas traditional therapies against cancer (chemotherapy and radiotherapy) have multiple limitations that lead to treatment failure and recurrence [48]. Moreover, EMT-targeting therapy may also be useful as a personalized medicine approach that complements conventional BC treatments. 

The evaluation of clinical samples revealed a vast amount of tumor heterogeneity. Such heterogeneity has crucial consequences in terms of adaptive drug resistance and tumor dormancy [45]. With these clinical samples, we also showed that BC cells with an intermediate EMT phenotype were enriched in the innermost outlet, a population affiliated with the CSC subtype. Although the current study is focused on BC samples, it may also be relevant to the detection of prostate cancer cells as some reports have suggested that prostate cancer cells can also be shed into the urine [49]. A combination of markers, including those specifically for bladder cells, could be implemented when realizing actual clinical utility to distinguish between various cancer types. CKs and genomic profiles associated with patient survival could also be used in combination to evaluate the association with clinicopathologic parameters and prognosis [50,51].

Future efforts may also be directed towards the design of primary trials to test the diagnostic capacity of our microfluidic device. Developing a method for early detection of new tumors and for effective surveillance for recurrences could reduce the morbidity of bladder cancer. Further improvements also include increasing the throughput of the spiral microfluidic device so that it is capable of processing a larger sample volume (e.g., > 100 mL) within a relatively short time.

## 4. Materials and Methods

### 4.1. Device Fabrication and Characterization

The silicon master was fabricated using standard microfabrication techniques, as described previously [52]: Six-inch-diameter silicon wafers were patterned using standard UV lithography and etched using deep reactive-ion etching (DRIE) to define the channels on the wafer (170 µm etch depth). After etching, the patterned silicon wafers were cleaned using acetone and isopropanol and treated with trichloro (1H, 1H, 2H, 2H perfluorooctyl) silane for 2 h to facilitate the polydimethylsiloxane (PDMS) mold release. After silanization, the mold was used to fabricate PDMS devices using standard soft lithography techniques. For standard soft lithography, the PDMS base and curing agent (Sylgard 184, Dow Corning Inc., Singapore, Singapore) were mixed in a 10:1 ratio and degassed for casting on the spiral microfluidic chip mold. The PDMS mixture was baked in an oven for 2 h at 70 °C. After curing, the PDMS was peeled from the mold, inlet and outlet holes were punched with the 1.5 mm Uni-Core^TM^ Puncher (Sigma-Aldrich Co. LLC., Singapore, Singapore) and the PDMS device was irreversibly bonded to Petri dishes covered with PDMS using an oxygen plasma machine (Diener Electronic, Ebhausen, Germany) to complete the channels. The settings for the plasma treatment were 21% pure oxygen and 21% air for 1 min 30 s. Then 15 cm and 12 cm long sections of TYGON flexible plastic tubing with an inner diameter of 0.06 IN were coupled to the fluidic inlets and outlets, respectively. Devices were first primed with 70% ethanol, followed by phosphate buffered saline (PBS), both at a flow rate of 1.7 mL/min.

The sorting devices were mounted on an inverted phase contrast microscope (Olympus IX71) coupled with a high-speed CCD camera (Phantom v9, Vision Research Inc., Wayne, NJ, USA). UMUC3 cells were spiked in PBS and pumped through the device using flexible Tygon^®^ tubing at different flow rates ranging from 0.5 mL/min to 2 mL/min. High-speed videos were captured using the Phantom Camera Control software and analyzed subsequently with the ImageJ software (Figure S3).

### 4.2. Cell Culture

UMUC3 bladder cancer cells were cultured in Minimum Essential Medium (MEM) (Thermofisher Scientific, Waltham, MA, USA) supplemented with 10% FBS (Invitrogen, Carlsbad, CA, USA) and 1% penicillin-streptomycin (Invitrogen, Carlsbad, CA, USA). Cells were cultured in a 5% CO_2_-humidified air atmosphere at 37 °C. They were passaged every 2–3 days at an 80% confluence. The medium was aspirated and the cultures were washed briefly with PBS. After removal of the PBS solution, 1 mL of trypsin was added and the cultures were placed in the incubator at a temperature of 37 °C during 5 min. Adherent cells were then gently dissociated from the flask bottom by inserting 1 mL of MEM media and diluted in a tube with 5 mL of media. After centrifugation at 1200 rpm for 3 min, the supernatant was aspirated and the remaining pellet was suspended in new culture flasks filled with 5 mL of MEM culture medium.

### 4.3. Characterisation of Sorted UMUC3 Cells 

For the evaluation of cell loss under various conditions, UMUC3 cells were harvested and stained with Hoechst. Cells were then collected from each outlet respectively, centrifuged down at 1200 rpm for 3 min and the supernatant was removed to a volume of 50 µL for imaging. The exact number of UMUC3 cells spiked was obtained by loading 50 µL of unprocessed cells to a 96-well plate, scanning the well under a fluorescent microscope and counting the cell number using ImageJ. The recovered UMUC3 cell number from each outlet was then compared with the initial spiked number to identify the cell loss. The error bars were determined with triplicate experiments. To examine the effects of viability after sorting, sorted cells were stained with 0.4% Trypan Blue (Thermofisher Scientific, USA) and viewed under a microscope. The percentage of viable cells was calculated both at the start and after 3 h.

### 4.4. Processing of Clinical Samples

This study was approved by respective institutional review boards (IRB) and the local ethics committee (National Healthcare Group (NHG)) (2016/00380). Informed and written consent was obtained from all patients. Seven patients with BC were recruited for this study (Table 1). Four samples (100 mL, 500 mL, 300 mL and 300 mL respectively) were collected from each patient during the process of trans-urethral removal of a bladder tumour (TURBT), each sample corresponding to one of the four steps of bladder washing processed (undiluted, first wash, second wash, third wash). Samples were collected in the morning and the entire processing was completed within 12 h. Samples were first concentrated to 25 mL and diluted with PBS in a 1:1 ratio. Diluted samples were further concentrated to 5 mL for the estimation of cell concentration with an automated cell counter. Samples were eventually resuspended into volumes to reach a cell concentration of 2.5 million per mL or below. BSA was added to achieve a concentration of 1.5% prior to filtration with the 40 µm cell strainer. Filtered cells were processed in the microfluidic spiral device. Outlets one, two and three were collected as a sample and outlets four and five are discarded as waste. The collected sample was split evenly in a 1:1 ratio for histopathology cytology and imaging, respectively.

### 4.5. Immunostaining of Sorted Cells

Cells collected from each of the outlets were stained with Hoechst 33,342 for enumeration. To determine the EMT phenotype, cells were also stained with anti-CK-FITC (130-098-802 Miltenyi Biotec 1:250) and anti-VIM-PE (30-106-369 Miltenyi Biotec 1:250) antibodies. 2.5% of BSA was added to avoid non-specific binding of the antibodies. To identify white blood cells, clinical samples were also stained with CD45-APC-Vio770 (130-113-677 Miltenyi Biotec 1:100). CK+ or VIM+ positive cells were counted as cancer cells only if they were also negative for CD45. Clinical samples were stained with the bladder cancer marker anti-survivin antibody (SAB5500179 SIGMA 1:100) and anti-EGFR antibody. Cells trapped by the filter were stained in situ with Hoechst 33,342 for 30 min (Figure S2), and subsequently imaged with a fluorescence microscope (Olympus IX81).

### 4.6. Imaging of Samples and Cell Counting

Imaging of the samples was done with 96-wells plates using a confocal microscope under 10× objective. An automated cell-counting algorithm was generated with an image processing software (ImageJ; Figure S8) to measure the cells counts, cell size distribution, cell intensity gradient and percentage of each phenotype. Fluorescence intensity was normalized relative to the background noise and expressed in arbitrary units (AU).

### 4.7. Wound Scratch Test 

Sorted cells from the first two outlets were split into two separate wells of a 48-well plate. After 2 to 4 h, cells were attached and a cross-directional scratch was created. Images were taken every 30 min for 7.5 h on four points of each scratch with a confocal microscope under optimal cell culture conditions.

### 4.8. Statistical Summary

Data were summarized as mean ± STD (Standard Deviation). Groups were compared using a t-test with a *p*-value of < 0.05 taken to reflect the presence of significant heterogeneity.

## 5. Conclusions

In summary, we demonstrated the detection of EBCCs using a microfluidic sorting device. Using a combination of filtration and inertial focusing principles, we were able to isolate enriched cells within hours, suggesting a potential utility for real-time detection. A range of characterization was applied to demonstrate the high efficiency of cancer cell recovery under the optimal flow rate and the specific retrieval of various EMT phenotype cell fractions from respective device outlets. The utility of this preclinical assay was validated with clinical samples. For routine screening of bladder cancer in clinics, the microfluidic sorting approach should be coupled to genomic analysis techniques such as digital PCR approaches to achieve the highest sensitivity of detection. These initial observations will open up new opportunities in the design of bladder cancer management and enhance personalized treatment.

## Figures and Tables

**Table 1:** Patient demographics. M = male, F = female, NMIBC = non-invasive muscle invasive bladder cancer, MIBC = muscle invasive bladder cancer.

No	Age/Sex	Cytology	Staging	Metastasis	Diagnosis
1	70/M	Inflammatory cells	cTaN0M0—Stage 0a	Nil	NMIBC
2	69/M	NA	cT4N3M1	Yes	MIBC—metastatic
3	89/M	NA	cT3N0M0	Nil	MIBC
4	84/M	Atypical, reactive—negative for HG malignant cells	cTaN0M0—Stage 0a	Nil	NMIBC
5	70/M	Highly atypical cells	cTaN0M0—Stage 0a	Nil	NMIBC
6	74/M	HG malignant cells seen	cT2N0M0	Nil	MIBC
7	70/M	Highly atypical cells, consistent with urothelial Ca	cT3N0M0	Nil	MIBC

## Supplementary Materials

The following are available online at https://www.mdpi.com/2072-6694/11/9/1274/s1, Figure S1: The recovery rate of EBCCs in the various versions of the sorting device, Figure S2: Filtering procedure prior to inertial microfluidic sorting, Figure S3: Flow rate experiment, Figure S4: Heterogeneity of the urine composition, Figure S5: Viability of UMUC3 cells, Figure S6: Effects of BSA addition on cell clumping, Figure S7: Identification of sorted bladder cancer cells, Figure S8: Automated cell-counting algorithm, Table S1: Current Methods of Bladder Cancer Diagnosis, Table S2: Recent Studies Focusing on Isolation of BC cells from Urine.

## Abbreviations

BC	Bladder Cancer
EBCCs	Exfoliated Bladder Cancer Cells
EGFR	Epidermal Growth Factor Receptor
SECs	Squamous Epithelial Cells
TURBT	Transurethral Resection of the Bladder Tumor
MIBC	Muscle-Invasive Bladder Cancer
NMBIC	Non-Muscle Invasive Bladder Cancer
PDMS	Polydimethylsiloxane
CTC	Circulating Tumor Cells
PBS	Phosphate Buffer Saline
EMT	Epithelial to Mesenchymal Transition
CSC	Cancer Stem Cell
