# Supplementary material for: Detection of Clinical Mesenchymal Cancer Cells from Bladder Wash Urine for Real-Time Detection and Prognosis
Source: Cancers (Basel). 2019 Aug 30;11(9):1274. doi: 10.3390/cancers11091274 (PMC6770607; doi:10.3390/cancers11091274)
Supplement: Supplementary file 1 [file cancers-11-01274-s001.pdf]

Supplementary Materials

Detection of Clinical Mesenchymal Cancer Cells from Bladder Wash Urine for Real-Time Detection and Prognosis

Bee Luan Khoo, Charlotte Bouquerel, Pradeep Durai, Sarannya Anil, Benjamin Goh, Bingcheng Wu, Lata Raman, Ratha Mahendran, Thomas Thamboo, Edmund Chiong, Chwee Teck Lim

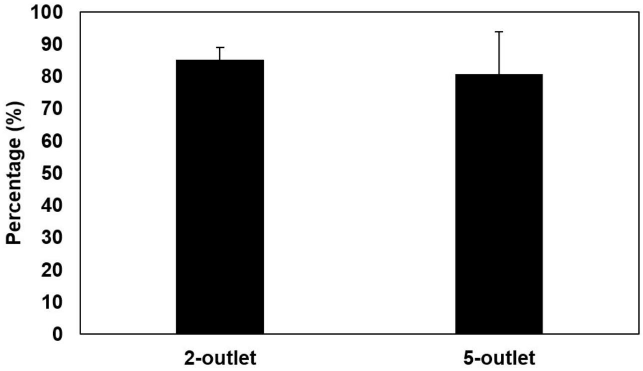

Figure S1. The recovery rate of EBCCs in the various versions of the sorting device. The dotted line serves as a reference to the recovery rate of current techniques of EBCC capture.

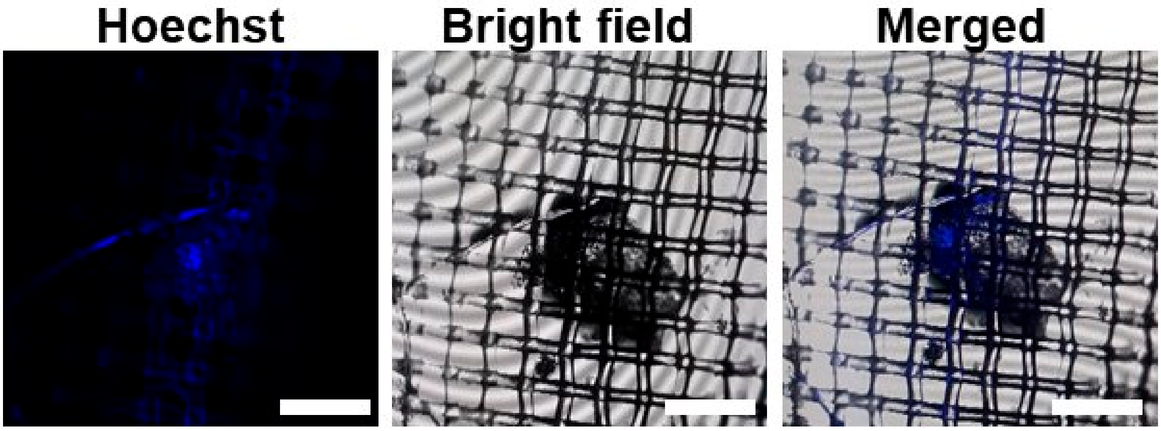

Figure S2. Filtering procedure prior to inertial microfluidic sorting. Immunostaining of the large squamous epithelial cells in situ. The epithelial cells were filtered out from the urine sample, but no UMUC3 cells were trapped, Scale bar is 100 μm.

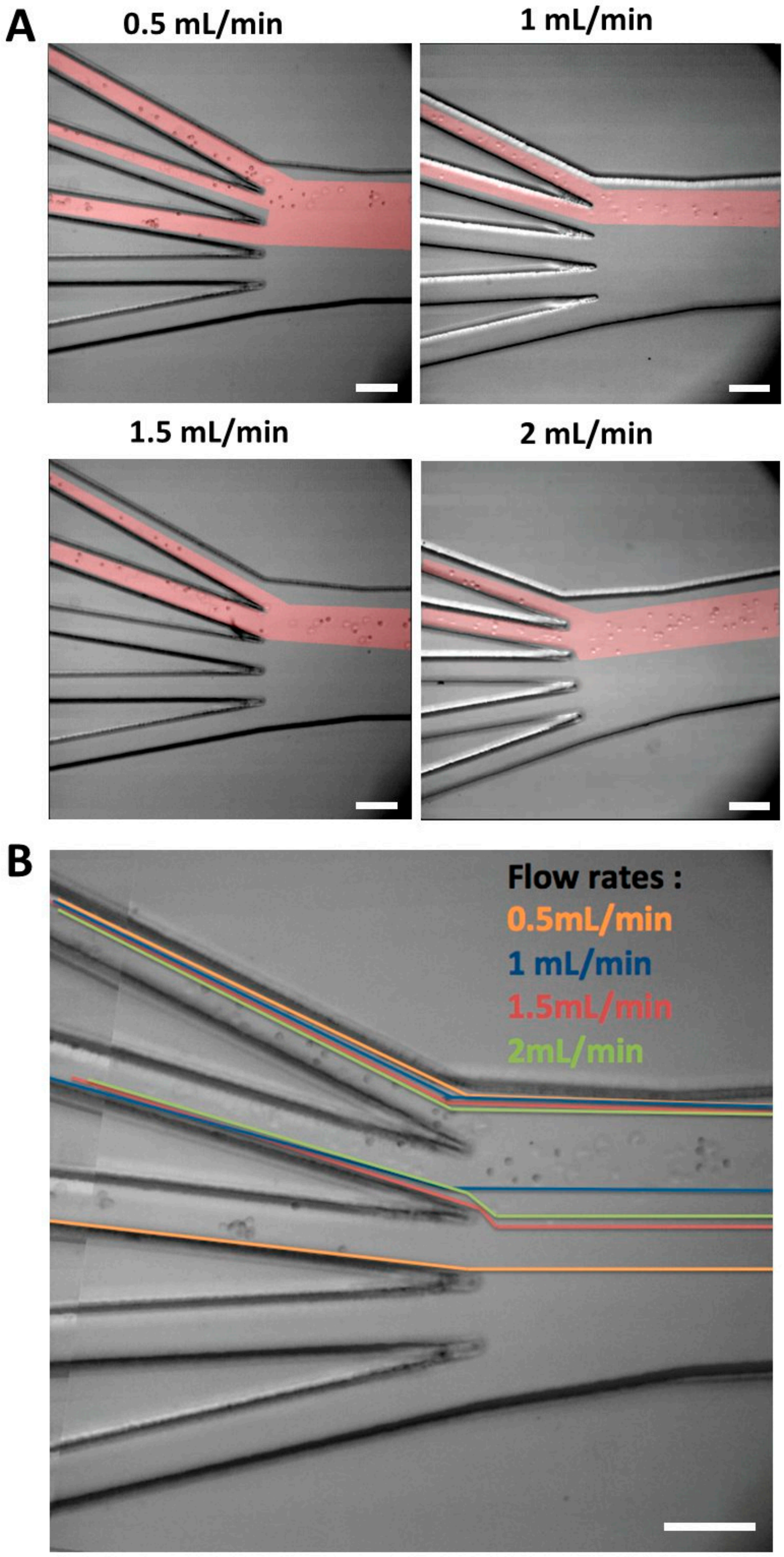

Figure S3. Flow rate experiment. (**A**) 4 flow rates were tested between 1 mL/min and 2 mL/min. (**B**) Merged cell stream for 4 flow rates. Scale bar is 200 μm.

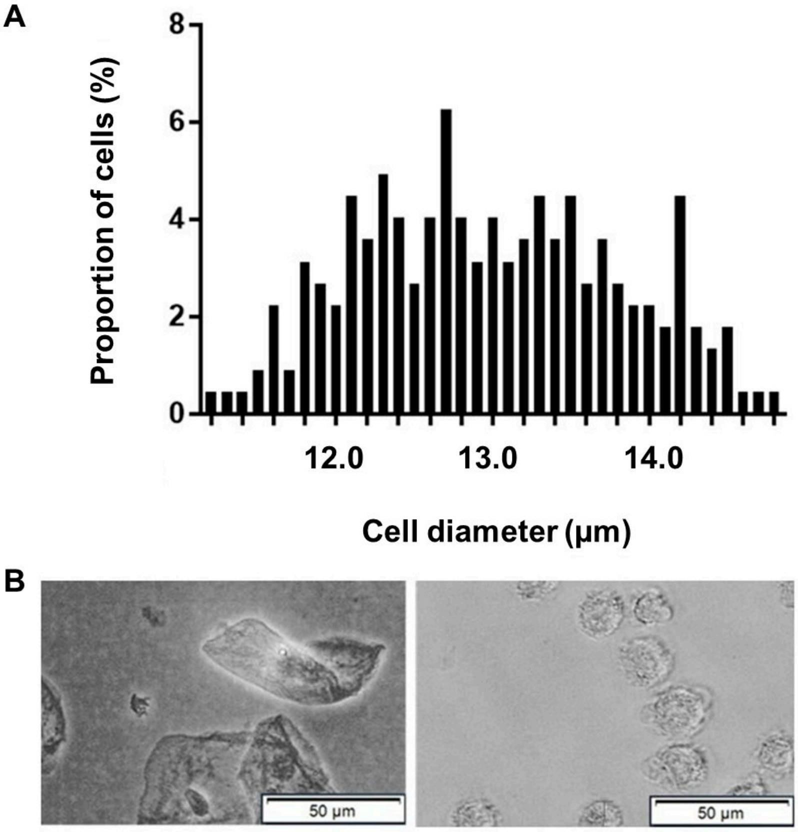

Figure S4. Heterogeneity of the urine composition. (**A**) UMUC3 cell size distribution profile determined by ImageJ software analysis. (**B**) Representative bright-field images of larger squamous epithelial cells (left) and smaller bladder cancer cells (right).

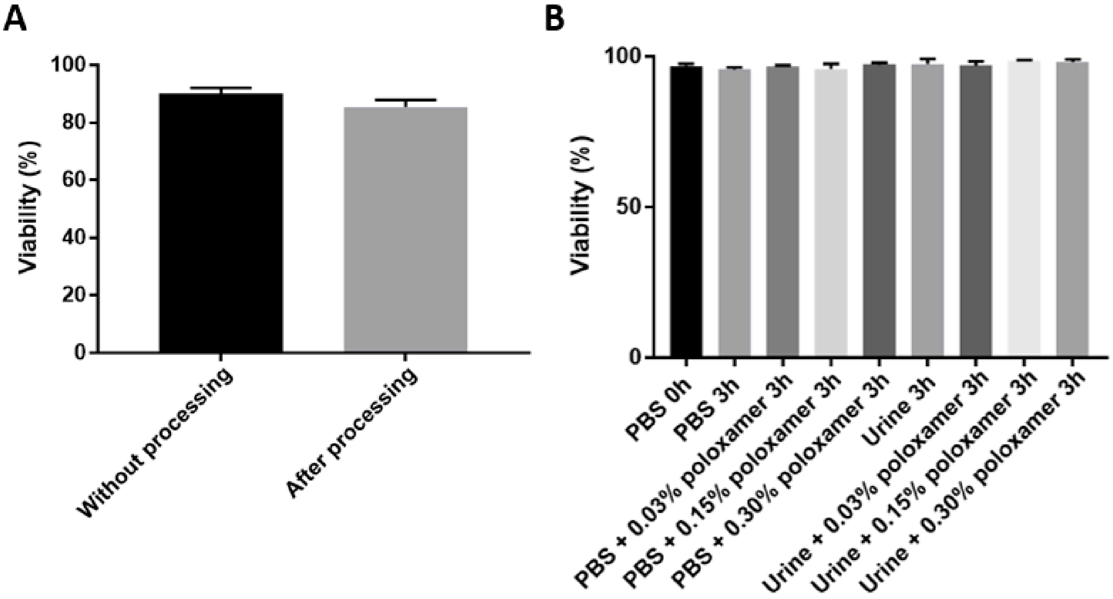

Figure S5. Viability of UMUC3 cells (**A**) With and without processing with spiral microchannel device (p-value = 0.07) (**B**) Under different Polaxamer concentrations after 3 h (p-value > 0.1).

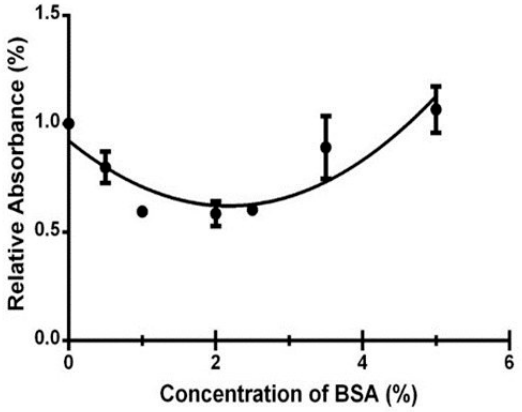

Figure S6. Effects of BSA addition on cell clumping. Changes in relative absorbance of each urine sample under the various concentration of BSA due to the presence of cell clumping.

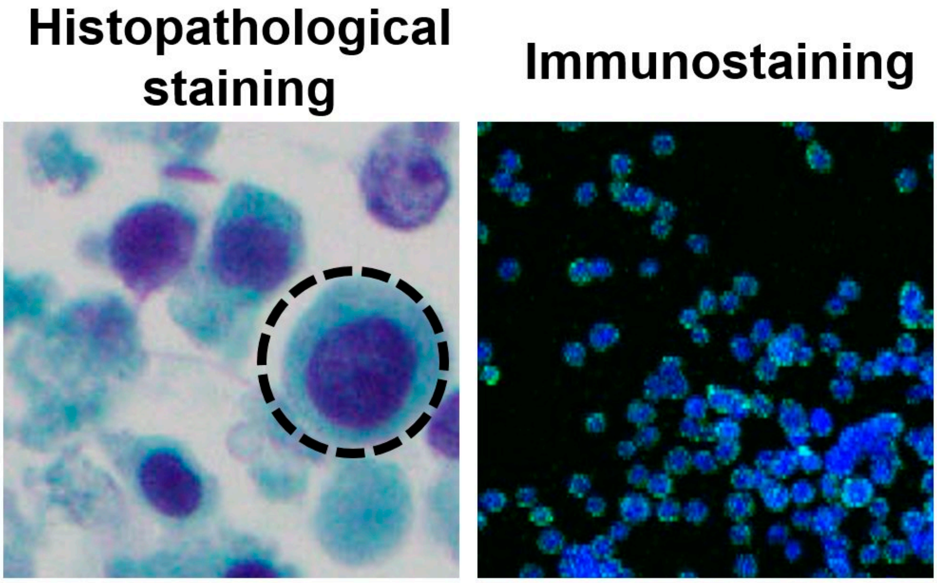

Figure S7. Identification of sorted bladder cancer cells. (Left) Nucleus/Cytoplasm (N/C) ratio and (right) histopathological staining or immunostaining.

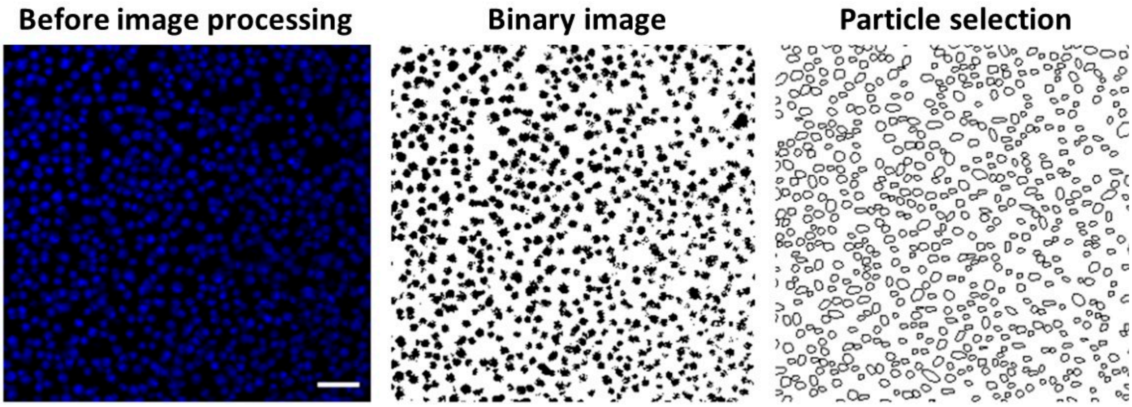

Figure S8. Automated cell-counting algorithm. Raw image (left), binary image after automated processing (centre) and selected cell particles by software algorithm (right).

Table S1. Current Methods of Bladder Cancer Diagnosis.

Options for BC Diagnosis	Description of Diagnosis Method
Urine cytology	Examine microscopically urinary sediment for the presence of tumor cells [1,2].
Cystoscopy	Detect growths in the bladder and determine the need for a biopsy or surgery with the use of cystoscopy [1,2].
TURBT	Remove a sample of the tumor for examination under a microscope [1,2].
Computed tomography (CT)	Show abnormalities or tumors in a detailed, cross-sectional view using X-ray. Measure the tumor's size [3].
Magnetic resonance imaging (MRI)	Produce detailed images of the tumor using a magnetic field. Measure the tumor's size [3].

Table S2. Recent Studies Focusing on Isolation of BC cells from Urine.

Technique	Description of Method	Sample	Performance
Filtration	Polycarbonate hydrophilic membrane filter of 8 μm pore [4]	Urine of 57 BC patients subjected to TURBT	Elimination of >99% smaller sized cells Recovery rate: 70%
	Parylene microfilter membrane (pores of 7.5 μm) sandwiched between two layers of polydimethylsiloxane [5]	54 urine and bladder wash samples	Specificity of 100% The sensibility of 53.3%
	Integrated microfiltration device and ELISA method [6]	35 BC patients and 20 healthy donors	Sensitivity of 77.1% Specificity of 90%
Immunocapture	Covalent binding of cancer-specific antibodies in microchannel [7]	Cancer cells spiked in patient urine with podocytes cell lines (negative control)	Selectivity of 99% The sensitivity of 100% sensitivity over a range of cell concentrations
	Use of antibody-modified hydroxyapatite (HAp) micro/nanostructured surfaces [8]	22 urine samples from bladder cancer patients	Capture efficiency of ~85%
Microarchitecture [9]	A label-free platform consisting of rows of posts with increasingly narrower gap widths to isolate BC cells according to their size and deformability	Spiking experiments of HT1376 bladder cancer cells with peripheral blood mononuclear cells 6 bladder washes from patients	Isolation efficiency of around 50% and an enrichment ratio of 22

References

1. Andersson, E.; Steven, K.; Guldberg, P. Size-based enrichment of exfoliated tumor cells in urine increases the sensitivity for DNA-based detection of bladder cancer. *PLoS ONE* **2014**, *9*, e94023.
2. Deng, Y.; Yi, L.; Lin, X.; Lin, L.; Li, H.; Lin, J.M. A non-invasive genomic diagnostic method for bladder cancer using size-based filtration and microchip electrophoresis. *Talanta* **2015**, *144*, 136–144.
3. Verma, S.; Rajesh, A.; Prasad, S.R.; Gaitonde, K.; Lall, C.G.; Mouraviev, V.; Aeron, G.; Bracken, R.B.; Sandrasegaran, K. Urinary bladder cancer: Role of MR imaging. *Radiographics* **2012**, *32*, 371–387.
4. Andersson, E.; Dahmcke, C.M.; Steven, K.; Larsen, L.K.; Guldberg, P. Filtration Device for On-Site Collection, Storage and Shipment of Cells from Urine and Its Application to DNA-Based Detection of Bladder Cancer. *PLoS ONE* **2015**, *10*, e0131889.
5. Birkhahn, M.; Mitra, A.P.; Williams, A.J.; Barr, N.J.; Skinner, E.C.; Stein, J.P.; Skinner, D.G.; Tai, Y.C.; Datar, R.H.; Cote, R.J. A novel precision-engineered microfiltration device for capture and characterisation of bladder cancer cells in urine. *Eur. J. Cancer* **2013**, *49*, 3159–3168.
6. Liang, L.; Wang, Y.; Lu, S.; Kong, M.; Lin, Y.; Cuzzucoli, F.; Wang, P.; Wang, S. Microchips for detection of exfoliated tumor cells in urine for identification of bladder cancer. *Anal. Chim. Acta* **2018**, *1044*, 93–101.
7. Macgregor-Ramiasa, M.; McNicholas, K.; Ostrikov, K.; Li, J.; Michael, M.; Gleadle, J.M.; Vasilev, K. A platform for selective immuno-capture of cancer cells from urine. *Biosens. Bioelectron.* **2017**, *96*, 373–380.
8. Zhang, W.; Zhao, K.; Banks, C.E.; Zhang, Y. Antibody-modified hydroxyapatite surfaces for the efficient capture of bladder cancer cells in a patient's urine without recourse to any sample pre-treatment. *J. Mater. Chem. B* **2017**, *5*, 8125–8132.

9. Abreu, C.F.M. Microfluidic Isolation and Characterisation of Bladder Cancer Cells from Urine for Early and Non-Invasive Diagnosis of Bladder Cancer. Master’s Thesis, Instituto Superior Técnico, Lisboa, Portugal, 2016.

© 2019 by the authors. Licensee MDPI, Basel, Switzerland. This article is an open access article distributed under the terms and conditions of the Creative Commons Attribution (CC BY) license (http://creativecommons.org/licenses/by/4.0/).
